# Comparison analysis of childhood body mass index cut-offs in predicting adulthood carotid intima media thickness: Tehran lipid and glucose study

**DOI:** 10.1186/s12887-021-02963-y

**Published:** 2021-11-06

**Authors:** Farhad Hosseinpanah, Amirhosein Seyedhoseinpour, Maryam Barzin, Maryam Mahdavi, Erfan Tasdighi, Pooneh Dehghan, Amin Momeni Moghaddam, Fereidoun Azizi, Majid Valizadeh

**Affiliations:** 1 Obesity Research Center, Research Institute for Endocrine Sciences, Shahid Beheshti University of Medical Sciences, Tehran, Iran; 2 Imaging Department, Taleghani Hospital, Shahid Beheshti University of Medical Science, Tehran, Iran; 3 Endocrine Research Center, Research Institute for Endocrine Sciences, Shahid Beheshti University of Medical Sciences, Tehran, Iran

**Keywords:** Obesity-pediatrics-adolescent-cardiovascular disease-subclinical atherosclerosis

## Abstract

**Background:**

The prevalence of obesity among children and adolescences have been increased, which can consequently increase the prevalence of metabolic and cardiovascular diseases later in life. The objective of this study is to compare the ability of different childhood body mass index cut-offs in prediction of carotid intima media thickness (CIMT) as an indicator of subclinical atherosclerosis.

**Methods:**

Participants were categorized into normal weight, overweight and obesity group, based on world health organization (WHO), center for disease control and prevention (CDC), international obesity task force (IOTF) and local IOTF cut-offs. After 18 years of follow up CIMT was measured. Akaike’s information criterion and relative efficiency were measured in order to compare regression models on the role of obesity on CIMT.

**Results:**

In this prospective cohort study, 1295 subjects aged 3 to 18 years old were enrolled. The overall prevalence of overweight was 15.4, 11.5, 16.3 and 14.1 along with obesity prevalence of 6.6, 8.5, 7.7 and 5.0% based on WHO, CDC, local IOTF and international IOTF criteria, respectively. CIMT was higher in obese compare to normal groups across all classification criteria. After regression analysis, international IOTF was the best to predict adulthood CIMT, followed by local IOTF and WHO. CDC had the least discriminatory ability.

**Conclusion:**

Due to the results of this study, IOTF could be a better tool in national and international surveillances of children in order to define overweight and obesity, which can help us to intervene more effectively in reducing the burden of cardiovascular diseases.

**Supplementary Information:**

The online version contains supplementary material available at 10.1186/s12887-021-02963-y.

## Background

In recent decades, the increasing prevalence of obesity showed concerning trends not only in developing countries, but also developed and low-income countries [1]. Along with epidemic trends of obesity in adults, obesity in childhood and adolescence was remarkably increased, which can consequently increase the prevalence of diabetes, cardiovascular disease, cancer and mortality later in life [2–4]. According to CDC and National Health and Nutrition Examination Survey (NHANES) reports, prevalence of obesity in children and adolescents in United States were 4 and 5% respectively in 1971, which increased to 12 and 17% in 2006 [5]. The prevalence of overweight and obesity among children and adolescences in Iranian national sample, reported in CASPIAN-V study, which was carried out from 2012 to 2015, was 9.4% and 11.4, respectively [6].

Carotid intima-media thickness (CIMT) evaluation is a noninvasive and sensitive technique in detection and quantification of subclinical atherosclerosis [7]. Also, CIMT is considered as a predictive factor of cardiovascular incidents. In the meta-analysis study performed in 2013, one standard deviation increase in CIMT, increased risk of myocardial infarction by 26% and stroke by 31% [8]. Also another study implied that one standard deviation increase in adolescence body mass index (BMI) was associated with 2.3 μm increase in CIMT in ages between 27 and 30 years [9].

BMI is the most commonly used index for screening and detection of obesity and overweight in clinical practice and epidemiological studies [10]. There are several reference data sets available as BMI cut-off values in children and adolescents, three of the most widely used are: 1- World Health Organization (WHO) using Z score and standard deviation based on multicenter data gathered from over one hundred countries and designed for two age ranges; 0–5 and 5–19 years old [11]; 2- United States Center for Disease Control and Prevention (CDC) based on age-gender specified BMI percentiles in Northern America [12]; and 3- International Obesity Task Force (IOTF), which defined BMI cut-off points based on data from six different countries by matching childhood BMI percentiles to adult cut-off values of 25 and 30 kg/m2 at the age of 18, using the Lambda-Mu-Sigma (LMS) method [13]. Although studies demonstrated the diversity in reported prevalence of overweight and obesity between aforementioned BMI definition systems [14], there is no consensus on determining the best system yet [15].

Screening and early diagnosis of high risk children and adolescents for cardiovascular disease is important in order to intervene effectively and lower the burden of the disease. In this study we aimed to compare the four childhood BMI systems (WHO, CDC and local IOTF and international IOTF) in predicting adulthood CIMT in order to suggest the best screening tool for cardiovascular disease.

## Methods

### Study population

Subjects of this cohort study were chosen from participants of Tehran Lipid and Glucose Study (TLGS), an ongoing community-based prospective study carried out to determine risk factors and outcomes of non-communicable diseases [16]. In the TLGS, 15,005 participants aged≥3 years were selected, using multistage random sampling method in district 13 of Tehran, capital of Iran, and were followed up every 3 years to update their demographics, clinical properties, biochemical status, anthropometric examinations and lifestyle data. The baseline cross-sectional survey was held from 1999 to 2001, a prospective follow-up surveys were conducted from 2002 to 2005 (phase II), 2006 to 2008 (phase III), 2009 to 2011 (phase IV), 2012 to 2015 (phase V) and 2016 to 2019 (phase VI).

**Fig. 1:**
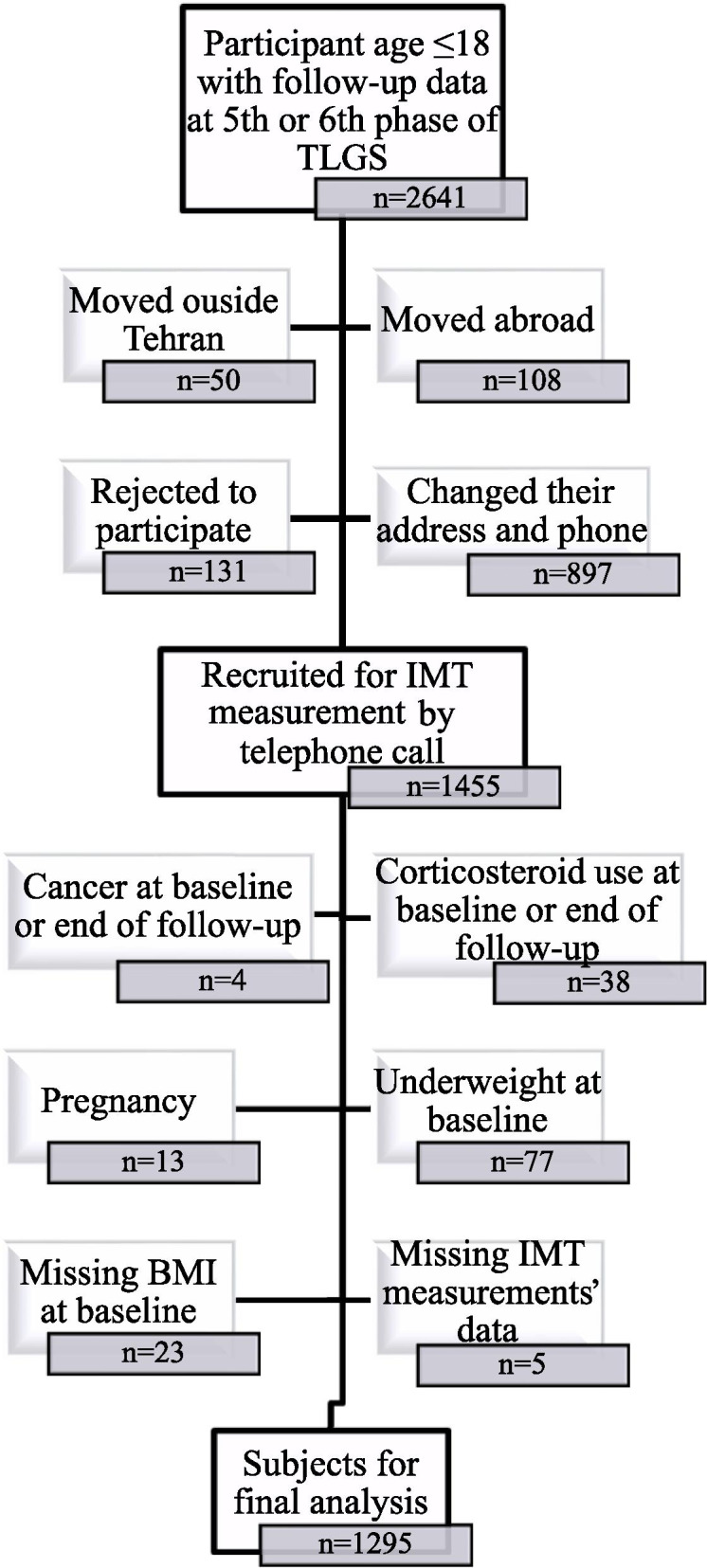
Flow chart of study participants

Of 2641 participants aged ≤18 years at the baseline with available data in 5th or 6th phase of the TLGS; we could recruit 1455 for CIMT measurement. After exclusion of subjects with cancer, pregnancy, chronic use of corticosteroid and extreme values of BMI (exceeding ±3SD), 1295 were enrolled (Fig. 1). The median follow up time was 18 years. CIMT measurement was conducted between Feb 2017 and Oct 2019.

### Anthropometric and laboratory assessment

Detailed information of TLGS protocol and laboratory procedures were published elsewhere [16]. Briefly, demographic and anthropometric data were obtained by trained health care professionals. Weight was measured to the nearest 100 g with digital electronic scale (Seca 707; range 0.1–150 kg, Hanover, MD, USA) while subjects were minimally clothed and barefoot. Height was measured in a standing position against wall, while shoulders are in normal alignment and without shoes; using tape stadiometer. Waist circumference (WC) measurement was performed utilizing non-flexible tape in the standing position at the narrowest level between iliac crest and lowest rib, without any pressure to the body’s surface, at the end of expiration. Measurements were recorded to the nearest 0.1 cm. BMI was calculated by the equation: *Weight*(*kg*)/〖*Height*(*m*)〗 ^ 2 . Systolic and diastolic blood pressures were measured two times with at least 30 s interval from right brachial artery at the heart level after 15 min of rest. The measurement was conducted by physician utilizing standard mercury sphygmomanometer (calibrated by Iranian Institute of Standards and Industrial Researches).

For fasting plasma glucose (FPG) and lipid concentrations measurements, a blood sample was obtained after a fasting period of 12 to 14 h, between 7 to 9 am at the TLGS Research Laboratory using commercially available laboratory kits (Pars Azmoon Inc., Tehran, Iran) adapted to a Selectra 2 auto analyzer. Samples were centrifuged within 30–45 min of collection. FPG and triglyceride were assessed by the enzymatic colorimetric method with glucose oxidase and glycerol phosphate oxidase respectively. Total cholesterol (TC) was also measured with cholesterol esterase and cholesterol oxidase using the enzymatic colorimetric method. After precipitation of apolipoprotein B containing lipoproteins with phosphotungistic acid, high-density lipoprotein cholesterol (HDL-C) was measured. The low density lipoprotein Cholesterol (LDL-C) was calculated by Friedwald formula if triglyceride (TG) concentrations were < 400 mg/dl [17]. The inter- and intra-assay coefficients of variation (CV) were both 2.2% for FPG; 0.6 and 1.6%, respectively for TG; and 0.5 and 2%, respectively for both HDL-C and TC [16].

### Assessment of the IMT of carotid artery

Two experienced radiologists measured the CIMT of extracranial carotid arteries using high resolution B-mode ultrasonography equipped with a linear 7.5–10 MHz transducer (Samsung Medison SonoAceR3 ultrasound machine). Subjects were positioned supine with neck extended and slightly rotated to the opposite side of examiner. The transverse plane scan throughout the course of the carotid artery was performed in order to assess the artery’s anatomy, locate atherosclerotic plaques (if there were any) and determine the site of maximal wall thickening in the near or far wall. The longitudinal scans in different angels were then performed. The measurements were implemented on optimal grey scale of the carotid artery, which is obtained in a plaque free arterial segment with the clear visualization of far wall arterial interface while luminal content is completely anechoic. The arterial lumen was placed in the center of the image while the focal zone was set to the level of arterial lumen by changing depth of the scan. The hypoechoic band between the echogenic surfaces of intima and adventitia of the arterial wall was considered as IMT. To assess CIMT, the investigators measured the distance of the first and second edge of the echogenic lines of the far wall. Left common carotid artery (LCCA) was gone through investigation in three locations and the average was considered as final measurement. Also sporadic measurement of the distal segment of both carotid arteries along with carotid bulb and internal carotid artery were performed.

In order to test the inter-observer agreement, both radiologists measured CIMT in a subsample of 30 participants, consists of 75% female with mean age of 38.5 ± 9.2 years and BMI of 25.2 ± 3.8 kg/m^2^. The interclass correlation coefficient (ICC) and 5% confident interval based on 2-way mixed-effects model were 0.79 and 0.55–0.90 respectively (ICC values between 0.75 and 0.9 indicate good reliability [18]).

### BMI definitions

Participants were divided into normal weight, overweight and obese groups based on the BMI z-scores specific for sex and age of the World Health Organization (WHO) standards and curves. All subjects were also reclassified according to the BMI criteria applied using Center for Disease Control and Prevention (CDC), International Obesity Task Force (IOTF) growth curves based international and local data obtained from TLGS study. Using 2007 WHO growth curves which is based on the 1971–1974 National Health and Examination Survey data, normal weight was defined as BMI z-score ≥ − 2 and ≤ + 1 SD; overweight as z-score > + 1 and ≤ + 2 SD which is equivalent to BMI 25 kg/m^2^ at 19 years; and obesity as z-score > + 2 SD which is equivalent to BMI 30 kg/m^2^ at 19 years [19]. According to 2000 CDC classification criteria based on the data from the Iranian national cross-sectional survey (CASPIAN study) on 21,111 subjects aged 6 to 18 years old, normal weight, overweight and obesity were considered as BMI z-scores ≥ − 1.65 and < + 1 SD; ≥ + 1 and < + 1.65 SD (percentiles ≥85th and < 95th); and ≥ + 1.65 SD (≥95th percentile) respectively [20]. As regards the IOTF criteria, the BMI cut points for normal weight, overweight and obesity were defined as when the BMI was equal to or greater than the value plotted on the sex related curves, derived from the study population based on Lambda-Mu-Sigma method, crossing a BMI of 18.5, 25 and 30 kg/m^2^ at the age of 18, respectively, which is referred as Local IOTF [13]. We also used predetermined international IOTF cut-offs, referred as international IOTF [13].

### Statistical analysis

Continuous variables were reported as mean ± SD or median (IQ 25–75) and Categorical variables were described as frequency percentages. Chi-square (or Fisher’s exact) tests were used to compare differences between sex groups. Four criteria were used to classify subjects as overweight or obese based on sex and age. WHO, CDC, IOTF criteria and local IOTF.

Centile curves for body mass index were developed by sex using the LMS method [21]. Figure 3 shows age-sex specific BMI percentiles (2nd, 9th, 25th, 50th, 75th, 91th and 98th) of study population based on local IOTF criteria. The sex specific z-score for BMI values equals to 25 and 30 kg/m^2^ at age 18 were obtained, using the equation:

$$M\ {\left(1+ LSz\right)}^{\frac{1}{L}}$$

As regards the CDC percentiles, the BMI ≥85th and < 95th CDC percentile of the reference population was considered as overweight and ≥ 95th percentile as obese. World Health Organization (WHO) Z scores were used to obtain obesity and overweight. Accordingly, the BMI ≥1 SD and < 2SD of the reference population was considered as overweight and ≥ 2SD of the reference population as obese. Also, percentile curves based on IOTF references that corresponded to cut-off points of 25 and 30 kg/m^2^ for adults were used to define overweight and obesity.

Kappa coefficient values were calculated in order to determine the rate of agreement between the cut-off points. Akaike’s information criterion (AIC) was used in order to evaluate the coefficients of different regression models on the role of obesity on IMT. Also relative efficiencies, as calculated by dividing the mean squared error of estimates of two models, were used to compare the efficiency of parameter estimates from models. All analyses were performed using STATA, and the LMS Chart Maker software package (version 2.0, 2005, London University, UK). Statistical significance was set at *P* < 0.05.

## Results

Among all 2641 subjects with follow-up data at 5th and 6th phase of TLGS, after exclusion, 1295 subjects aged 3 to 18 years, 670 boys (51.7%) and 625 girls (48.3%), were enrolled. Baseline characteristics of study subjects and lost to follow-up group were compared, and the results suggested that in spite of statistically significant difference in terms of some variables; there is no clinically significant difference between the characteristics of follow-up and lost to follow-up group and the final results of the study subjects can be representative of the whole population of the TLGS (Supplementary Table 2). Baseline characteristics and cardio-metabolic profile of the participants are demonstrated in Table 1. Among all cardio-metabolic risk factors at baseline, the differences between normal weight, overweight and obesity group were significant, except for FPG in CDC BMI classification system. Moreover, all cardio-metabolic risk factors in obese groups were significantly higher than normal weight group. Across all classification systems there were significant differences between overweight and normal weight groups except HTN in all classification systems and FPG in local IOTF. The prevalence of family history of CVD was different between normal weight, overweight and obese groups across all classification systems.

**Table 1:** Baseline characteristic of the participants

	WHO			CDC			Local IOTF			International IOTF		
	Normal weight	Overweight	Obese	Normal weight	Overweight	Obese	Normal weight	Overweight	Obese	Normal weight	Overweight	Obese
**N** (%)	1011 (78.1)	199 (15.4)	85 (6.6)	889 (79.7)	128 (11.5)	98 (8.5)	984 (76)	211 (16.3)	100 (7.7)	1048 (80.9)	182 (14.1)	65 (5.0)
**Male** (%)	523 (51.7)	96 (48.2)	51 (60)	463 (52.1)	60 (46.9)	55 (56.1)	513 (52.1)†	94 (44.5)	63 (63)	545 (52.0)	88 (48.4)	37 (56.9)
**Age** (years)	10.7 ± 4.1†	11.9 ± 3.6*	11.1 ± 3.7	11.9 ± 3.1†	12.3 ± 3.1	13.0 ± 3.0**	10.8 ± 4.1	11.4 ± 4.1	11.4 ± 3.6	10.7 ± 4.1†	12.3 ± 3.4*	11.1 ± 3.8
**Weight** (kg)	36.5 ± 14.9†	52.4 ± 18.2*	59.8 ± 21.1**	40.2 ± 13.2†	54.0 ± 15.7*	67.9 ± 15.6**	36.5 ± 14.7†	49.7 ± 19.3*	60.4 ± 20.2**	36.6 ± 14.9†	55.5 ± 17.2*	62.1 ± 21.7**
**BMI** (kg /m^2^)	17.2 ± 2.5†	22.5 ± 3.2*	27.3 ± 6.5**	17.70 ± 2.5†	22.8 ± 2.4*	28.3 ± 5.3**	17.1 ± 2.5†	21.9 ± 3.3*	26.9 ± 6.1**	17.3 ± 2.6†	23.4 ± 2.9*	28.3 ± 6.9**
**WC** (cm)	61.2 ± 8.6†	73.1 ± 10.1*	81.6 ± 13.9**	62.5 ± 8.1†	74.4 ± 8.3*	83.5 ± 11.6**	61.1 ± 8.4†	72.4 ± 10.1*	80.9 ± 13.3**	61.4 ± 8.6†	75.0 ± 9.6*	82.9 ± 14.7**
**Abdominal obesity** (%)	23 (2.7)†	67 (36.6)*	66 (85.7)**	29 (3.3)†	49 (38.3)*	78 (79.6)**	21 (2.5)†	60 (33.0)*	75 (81.5)**	27 (3.1)†	76 (44.4)*	53 (89.8)**
**SBP** (mmHg)	102.6 ± 11.3†	107.5 ± 12.2*	110.9 ± 11.9**	103.5 ± 10.8†	107.9 ± 12.5*	112.6 ± 11.9**	102.7 ± 11.3†	106.2 ± 12.1*	111.6 ± 12**	102.8 ± 11.4†	107.1 ± 11.5*	112.7 ± 11.6**
**DBP** (mmHg)	69.4 ± 9.5†	72.6 ± 8.7*	73.9 ± 10.7**	70.0 ± 9.4†	72.9 ± 8.8*	73.9 ± 10.4**	69.4 ± 9.5†	71.9 ± 8.9*	74.3 ± 10.6**	69.6 ± 9.6†	72.3 ± 9.0*	75.0 ± 9.9**
**Hypertension** (%)	144 (14.6)†	32 (16.3)	27 (32.1)**	105 (12.0)†	21 (16.7)	21 (21.4)**	138 (14.4)†	34 (16.5)	31 (31.3)**	154 (15.1)†	27 (15.0)	22 (33.8)**
**FPG** (mg/dl)	86.86 ± 8.28†	88.8 ± 7.7*	90.2 ± 7.5**	87.8 ± 7.9	88.7 ± 7.2	89.6 ± 8.1	86.9 ± 8.2†	87.9 ± 8.0	90.6 ± 7.7**	86.9 ± 8.3†	89.0 ± 7.8*	90.60 ± 7.42**
**TC** (mg/dL)	166.5 ± 30.2†	179.5 ± 31.0*	185.6 ± 38.1**	166.1 ± 30.7†	179.8 ± 31*	186.4 ± 37.4**	166.1 ± 30.2†	180.2 ± 30.8*	183.6 ± 37.1**	166.8 ± 30.2†	179.1 ± 30.5*	189.7 ± 41.6**
**TG** (mg/dl)^a^	87.5 (67–116)†	112 (86–154.5)*	145 (91–187)**	89 (67–117)†	112 (86.5–154.5)*	151.5 (98.8–195.3)**	87 (66–115)†	110 (86–151.5)*	138 (89.5–181)**	88 (67–116)†	113.5 (86.8–161)*	146 (97.5–188.5)**
**HDL-C** (mg/dl)	45.1 ± 10.9†	41.4 ± 9.5*	40.2 ± 8.9**	45.1 ± 10.8†	40.6 ± 8.8*	39.3 ± 9.5**	45.2 ± 10.8†	41.7 ± 10*	40.0 ± 8.6**	45.1 ± 10.8†	40.7 ± 9.4*	40.3 ± 8.9**
**LDL-C** (mg/dl)	101.6 ± 26.9†	112.4 ± 27.8*	116.5 ± 34.0**	101.1 ± 27.4†	113.7 ± 27.3*	115.5 ± 33.6**	101.4 ± 26.9†	113.1 ± 27.3*	114.6 ± 33.0**	101.9 ± 26.9†	111.8 ± 26.9*	119.8 ± 37.5**
**Family history CVD** (%)	31 (3.1)†	14 (7.1)*	2 (2.4)	30 (3.4)†	12 (9.5)*	3 (3.1)	30 (3.1)†	14 (6.7)*	3 (3.1)	32 (3.1)†	13 (7.3)*	2 (3.1)

*WHO* World Health Organization, *CDC* Center for Disease Control and Prevention, *IOTF* International Obesity Task Force, *CVD* Cardio vascular disease, *BMI* Body mass index, *WC* Waist circumference, *SBP* Systolic blood pressure, *DBP* Diastolic blood pressure, *TC* Total cholesterol, *TG* Triglycerides, *HDL-C* High-density lipoprotein cholesterol, *LDL-C* Low-density lipoprotein cholesterol

Data are given as the mean ± SD or median (IQ 25–75) unless otherwise indicated (^a^)

*P value< 0.05 comparison between Normal weight and Over weight

** *P* value< 0.05 comparison between Normal weight and Obese

† *P* ANOVA < 0.05

**Fig. 2:**
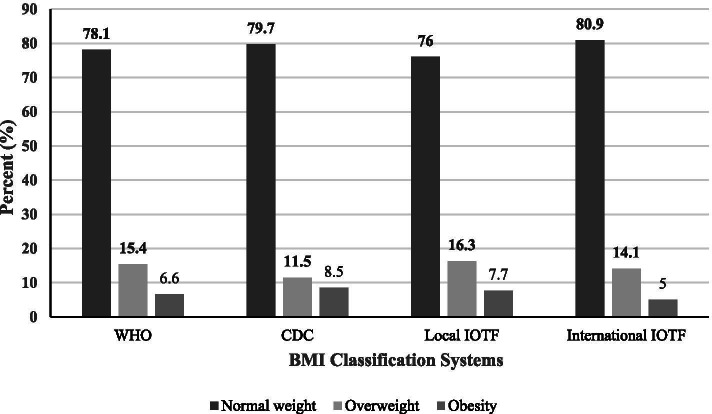
Differences in BMI classification according to World Health Organization (WHO), Center for Disease Control and Prevention (CDC), local International Obesity Task Force (IOTF) and international IOTF criteria

The overall prevalence of overweight was 15.4, 11.5, 16.3 and 14.1 along with obesity prevalence of 6.6, 8.5, 7.7 and 5.0% based on WHO, CDC, local IOTF and international IOTF criteria, respectively. More Children were classified in overweight or obesity group using local IOTF in contrast to WHO, CDC and international IOTF criteria, while international IOTF had the least numbers (Fig. 2).

The kappa correlation coefficient of international IOTF with local IOTF, WHO and CDC were 0.787, 0.867 and 0.821, respectively. Also the kappa correlation coefficient value was 0.889 between local IOTF and WHO; 0.812 between local IOTF and CDC; and 0.815 between WHO and CDC.

**Fig. 3:**
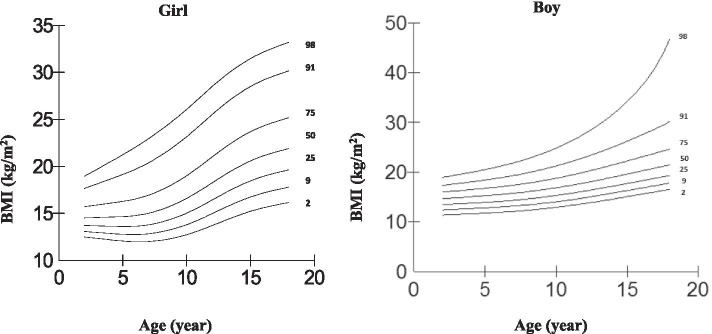
Age-sex specific BMI percentiles (2nd, 9th, 25th, 50th, 75th, 91th and 98th) of study population based on IOTF criteria

We plotted age-sex specific BMI percentile curves according to IOTF BMI classification criteria using LMS regression method which is presented in Fig. 3. Age-sex specific cut-off values for overweight and obesity, equals to BMI 25 and 30 kg/m^2^ at the age of 18 were calculated, which is presented in Supplementary Table 1.

Participant’s characteristics at the end of follow up were also presented in Table 2. CIMT was significantly higher in obese than normal weight groups in all four BMI classification systems. However, only in International IOTF classification CIMT in overweight group was significantly higher than normal weight. Among cardio-metabolic risk factors at the end follow-up, only total cholesterol and low density lipid cholesterol (LDL-C) were not significantly different between BMI categories in all four BMI classification systems. Also FPG’s difference in CDC, local IOTF and international IOTF classification system did not reach the significance level.

**Table 2:** Participants’ characteristic at the end of the follow-up

	WHO			CDC			Local IOTF			International IOTF		
	Normal weight	Overweight	Obese	Normal weight	Overweight	Obese	Normal weight	Overweight	Obese	Normal weight	Overweight	Obese
**Age at IMT measurement** (years)	29.6 ± 4.1†	30.6 ± 3.7*	29.8 ± 3.7	30.7 ± 3.2†	31.1 ± 3.3	31.6 ± 3.2**	29.6 ± 4.1	30.1 ± 4.2	30.1 ± 3.5	29.5 ± 4.1†	31.1 ± 3.4*	29.8 ± 3.9
**Weight** (kg)	72.2 ± 14.9†	83.2 ± 17*	92.5 ± 23.12**	73.1 ± 14.9†	83.0 ± 16.2*	91.6 ± 22.8**	72.0 ± 14.8†	82.2 ± 16.7*	92.3 ± 22.2**	72.6 ± 15.2†	83.5 ± 16.9*	94.3 ± 24.1**
**BMI** (kg /m^2^)	25.2 ± 3.99†	29.1 ± 4.3*	32.5 ± 7.0**	25.5 ± 3.9†	29.1 ± 3.9*	32.3 ± 6.6**	25.1 ± 3.9†	28.9 ± 4.4*	32.0 ± 6.7**	25.3 ± 4.1†	29.3 ± 4.3*	33.2 ± 7.4**
**WC** (cm)	86.5 ± 10.8†	94.9 ± 12.4*	100.8 ± 16.1**	87.4 ± 10.6†	95.3 ± 12.3*	100.5 ± 15.6**	86.4 ± 10.7†	93.9 ± 12.6*	100.5 ± 15.5**	86.7 ± 10.9†	95.1 ± 12.4*	102.3 ± 16.8**
**Abdominal obesity** (%)	366 (36.8) †	122 (63.2) *	67 (79.8)**	346 (39.6)†	82 (66.7) *	72 (74.2)**	354 (36.6) †	125 (61.3)*	76 (76.8)**	378 (37.6)	117 (66.1)*	51 (79.7) **
**SBP** (mmHg)	106.7 ± 12.0†	107.9 ± 11.7	113.7 ± 14.1**	106.8 ± 11.9†	107.1 ± 11.6	113.6 ± 14.1**	106.7 ± 11.9†	107.5 ± 12.2	113.8 ± 13.5**	106.8 ± 12.0†	107.9 ± 11.5	114.9 ± 14.5**
**DBP** (mmHg)	72.5 ± 8.9†	73.4 ± 9.5	75.6 ± 12**	72.7 ± 8.8†	73.0 ± 9.8	76.2 ± 11.9**	72.5 ± 8.9†	72.9 ± 9.4	75.9 ± 11.6**	72.5 ± 8.9†	73.5 ± 9.8	76.0 ± 12.4**
**Hypertension,** n(%)	35 (3.7)†	9 (4.8)	10 (13.3) **	30 (3.6)†	6 (5.0)	12 (13.2)**	34 (3.7)†	9 (4.5)	11 (12.2)**	38 (3.8)†	8 (4.7)	8 (14.0)**
**FPG** (mg/dl)	88.5 ± 9.1†	89.9 ± 11.6	90.7 ± 13.7	88.6 ± 10.0	89.7 ± 9.5	90.8 ± 13.2	88.5 ± 9.1	89.5 ± 11.6	90.3 ± 12.9	88.5 ± 9.2	89.9 ± 11.7	90.6 ± 14.8
**TC** (mg/dL)	172.2 ± 33.6	176.5 ± 33.4	174.7 ± 35.6	174.4 ± 33.6	176.1 ± 34.5	177.1 ± 36.9	172.1 ± 33.7	176.3 ± 31.5	175.5 ± 37.5	172.6 ± 33.4	173.3 ± 34.7	179.1 ± 35.6
**TG** (mg/dl)^a^	94 (68–138.5)†	108 (79–154)*	107.5 (75.3–159.8)	98 (70–142)†	107 (73–154)	120 (83.5–171)**	94 (68–139)†	106.5 (79–154) *	108 (76–156) **	95 (69–139) †	105 (73.3–151)*	114.5 (78.3–172.5)**
**HDL-C** (mg/dl)	48.2 ± 11.1†	45.2 ± 10.1*	45.4 ± 10.2	47.8 ± 11.1†	45.4 ± 10.2	44.4 ± 10.7**	48.3 ± 11.0†	45.4 ± 10.6*	45.1 ± 10**	48.1 ± 11.0†	44.9 ± 10.1*	45.2 ± 10.5
**LDL-C** (mg/dl)	101.2 ± 28.1	105.8 ± 29.7	103.9 ± 32.3	103.3 ± 28.2	104.8 ± 30.6	105.4 ± 33.1	101.0 ± 28.2	105.6 ± 27.7	104.8 ± 33.8	101.6 ± 28.0	103.2 ± 30.4	107.3 ± 33.4
**Smoking,** n(%)	173 (17.7) †	47 (24.1)	24 (29.3)**	159 (18.3) †	32 (25.4)	28 (28.9)**	169 (17.8) †	47 (22.9)	28 (28.9)**	182 (18.0) †	43 (23.9)	19 (30.6)**
**CIMT** (mm)	0.55 ± 0.1†	0.56 ± 0.1	0.58 ± 0.1**	0.55 ± 0.09†	0.56 ± 0.09	0.59 ± 0.1**	0.55 ± 0.09†	0.56 ± 0.09	0.58 ± 0.1**	0.55 ± 0.09†	0.57 ± 0.1*	0.59 ± 0.1**

*WHO* World Health Organization, *CDC* Center for Disease Control and Prevention, *IOTF* International Obesity Task Force, *HDL-C* High-density lipoprotein cholesterol, *LDL-C* Low-density lipoprotein cholesterol, *SBP* Systolic blood pressure, *DBP* Diastolic blood pressure, *CIMT* Carotid Intima Media Thickness

Data are given as the mean (SD) or median (IQ 25–75) unless otherwise indicated (^a^)

* *P* value< 0.05 comparison between Normal weight and Over weight

** *P* value< 0.05 comparison between Normal weight and Obese

† *P* ANOVA < 0.05

Multiple linear regression analysis was performed before and after adjustment for baseline demographics, family history of cardiovascular disease, smoking and adulthood BMI in order to discriminate the best model fit for adulthood CIMT prediction (Table 3). The Akaike’s Information Criterion (AIC) was calculated for each model, of which lower AIC values represents better fit. AIC values of international IOTF were the lowest among four BMI classification in unadjusted and all adjusted models. Local IOTF and WHO had the second and third low values of AIC, respectively, while CDC had the highest values of AIC in all models. After adjustment for adulthood BMI (model 3), although the comparison between AIC values of different BMI classifications did not change, the difference between AIC values of WHO, local IOTF and international IOTF decreased.

**Table 3:** Multiple linear regression results of WHO, CDC, Local IOTF and International IOTF with CIMT

		WHO			CDC			Local IOTF			International IOTF		
		β	SE	AIC	β	SE	AIC	β	SE	AIC	β	SE	AIC
**Unadjusted model**													
	**Overweight**	0.01544	0.00736	− 2419.43	0.00784	0.00902	− 2071.13	0.01347	0.00720	− 2420.09	0.01831	0.00761	− 2424.17
	**Obesity**	0.03455	0.01072		0.04062	0.01016		0.03454	0.00996		0.04522	0.01212	
**Adjusted models**													
**Model 1**	**Overweight**	0.01226	0.00727	− 2467.49	0.00546	0.00889	− 2105.46	0.00974	0.00708	− 2468.98	0.01447	0.00755	− 2471.04
	**Obesity**	0.03682	0.01053		0.04021	0.01005		0.03744	0.00979		0.04618	0.01190	
**Model 2**	**Overweight**	0.01431	0.00744	− 2358.31	0.00626	0.00913	− 2032.45	0.01150	0.00729	− 2359.50	0.01618	0.00771	− 2362.02
	**Obesity**	0.03669	0.01091		0.03949	0.01022		0.03732	0.01010		0.04689	0.01234	
**Model 3**	**Overweight**	0.00014	0.00774	− 2381.21	−0.00880	0.00945	− 2050.14	−0.00292	0.00761	− 2382.40	0.00216	0.00801	−2382.88
	**Obesity**	0.01268	0.01165		0.01787	0.01105		0.01488	0.01082		0.022252	0.01309	

*WHO* World Health Organization, *CDC* Center for Disease Control and Prevention, *IOTF* International Obesity Task Force, *SE* Standard Error, *AIC* Akaike information criterion

Model 1: Adjusted for age, gender; Model 2: Model 1 + family history of cardiovascular disease, smoking; Model 3: Model 2 + adulthood BMI

Relative efficiency (RE) was calculated for comparing WHO, CDC, local IOTF and International IOTF cut-offs in discrimination of adulthood CIMT (Table 4). RE values of local IOTF, WHO and CDC with international IOTF were > 1 in all models. This indicate that international IOTF curves discriminate adulthood CIMT better than the other three. Also, RE values of local IOTF with WHO and CDC were < 1, which represent that local IOTF is more efficient than WHO and CDC. Also WHO was more efficient in adulthood CIMT discrimination than CDC, while the RE values of WHO and CDC were > 1.

**Table 4:** Relative efficiency of Local IOTF, CDC, WHO and International IOTF (Row/Column)

	International IOTF				WHO				CDC			
	Unadjusted Model	Model 1	Model 2	Model 3	Unadjusted Model	Model 1	Model 2	Model 3	Unadjusted Model	Model 1	Model 2	Model 3
**Local IOTF**	1.0031	1.0016	1.0020	1.0004	0.9995	0.9988	0.9990	0.9990	0.9891	0.9825	0.9827	0.9821
**CDC**	1.0142	1.0194	1.0197	1.0186	1.0105	1.0166	1.0166	1.0173				
**WHO**	1.0037	1.0027	1.0030	1.0013								

*WHO* World Health Organization, *CDC* Center for Disease Control and Prevention, *IOTF* International Obesity Task Force

Model 1: Adjusted for age, gender; Model 2: Model 1 + family history of cardiovascular disease, smoking; Model 3: Model 2 + adulthood BMI

## Discussion

This study investigated the ability of commonly used childhood BMI cut-offs to discriminate adulthood CIMT as a contributor of subclinical atherosclerosis. Our results demonstrated that between WHO, CDC, international IOTF and local IOTF cut-offs, international IOTF is the best to predict adulthood CIMT, followed by local IOTF and WHO. CDC had the least discriminatory ability.

The BMI cut-off values to define overweight and obesity are fixed in adults, which are 25 and 30 kg/m^2^ respectively. However, in children different cut-off values have been presented, which are based on Z-scores and percentiles obtained from different data sets [15]. WHO, CDC and IOTF are the most commonly used BMI systems in childhood and adolescence. Utilizing different BMI systems results in diverse outcomes on epidemiology of overweight and obesity with the different sensitivity and specificity [20, 22]. In our study, the highest prevalence of overweight/obesity was observed in local IOTF (24%), while in international IOTF had the lowest (19%). Though, the kappa agreement between all four discussed BMI definitions were good in our study.

Overweight and obesity during childhood and adolescence are linked to cardiovascular and metabolic adverse effects in adulthood, which include hypertension, dyslipidemia, insulin resistance, adult morbidity and premature mortality [23, 24]. The result of our study demonstrated that almost all cardio-metabolic risk factors, both at baseline and end of follow-up, were significantly different between normal weight, overweight and obese in all four aforementioned BMI definitions. Thus, defining best BMI cut-offs for predicting adulthood clinical outcomes and risk factors should be focused.

CIMT is considered as a contributor of subclinical atherosclerosis and a predictive factor of cardiovascular incidence [8]. The results of previous studies indicate that childhood overweight or obesity predicts an increased risk of thicker CIMT during adulthood [25, 26]. However, in the study of Lee et al., the association between obesity in adolescence and increased CIMT in adulthood was only observed among male subjects [27]. In our study, CIMT was significantly different among normal weight, overweight and obese category in all for BMI systems. Along with childhood obesity, other cardiovascular risk factors during childhood were previously demonstrated to be associated with adulthood CIMT. Xi and colleagues indicated the effect of childhood hypertension on adulthood CIMT [28]. Furthermore, hypertension, dyslipidemia and metabolic syndrome during childhood and adolescence were related to thicker CIMT in adulthood [26, 29].

We utilized CIMT as a subjective and sensitive contributor of subclinical cardiovascular disease in order to discriminate different childhood BMI definitions in prediction of cardiovascular disease. The same approach was presented in the study of Fan et al. in order to discriminate different childhood hypertension definition [30]. However, in most of the previous studies, other cardio-metabolic risk factors such as blood pressure, laboratory findings, anthropometric variables and metabolic syndrome were used as outcome variable [31–36].

The results of our study indicated that international IOTF, followed by local IOTF had the best ability to discriminate adulthood CIMT, WHO placed next, with CDC having the least discriminatory ability. Although several studies compared BMI classifications, there is no study that use similar approach using IMT to compare different BMI classifications. Corresponding to our results, one study yielded that local IOTF better discriminated cardio metabolic risk factors than local CDC [31] and another study showed that the specificity of IOTF in identifying children with obesity by clustered cardio metabolic risk factor placed above WHO, while WHO is more sensitive [34]. Furthermore, the difference in discriminatory ability of the local IOTF, international IOTF and WHO became less significant after adjustment for adulthood BMI, while CDC was still significantly less powerful. The study of Martinez-Costa, in which the difference between WHO, CDC and Spanish reference criteria were investigated, WHO better discriminated between overweight and obesity in metabolic and vascular comorbidities [33]. Moreover, another study yielded that WHO is more sensitive but less specific than CDC in discrimination of cardio metabolic risk in children with overweight, although their ability were similar in children with obesity [36]. In contrary, Lee et al. found that CDC have better discriminatory ability to identify components of metabolic syndrome than WHO [35].

Along with the question of which BMI definition fits best, another question is whether international or local references are better. Using international BMI cut-points rather than local references, make it possible to have the same language in interpretation of obtained data from different countries and to compare them easily. Although, the applicability of international references is under question, as observed in previous studies [22, 34]. In this study, we used both international IOTF references, derived from six countries, and local IOTF cut-offs, which is obtained from our study population. The results demonstrated that international IOTF had better discriminatory ability of future CIMT compared to local IOTF. This result may be due to the fact that our study was representative of the population of Tehran, the metropolitan capital of Iran, but not nationally representative. Therefore it would be better to determine local IOTF cut-offs by using national data.

Regarding the limitations of this study, some variables with the possible confounding effect like socioeconomic status, dietary habits, physical activity and BMI changes between stages were not taken into account. Also the data were obtained from a metropolitan city of Iran and may not be nationally representative. Using CIMT as an outcome variable, prospective design of the study, duration of follow up, using subjective variables and also using different statistical approaches to compare different BMI criteria were the points of strength of the study.

In conclusion, our study resulted that IOTF cut-offs in children is a better tool for prediction of adulthood subclinical atherosclerosis, compared to WHO and CDC, while CDC has the least discriminatory ability. These results suggest that IOTF could be a better tool in national and international surveillances of children in order to define overweight and obesity, which can help us to intervene more effectively in reducing the burden of cardiovascular diseases.

## Supplementary Information

**Additional file 1: Supplementary Table 1**. Cut-Off Values for BMI for Overweight and Obesity According to Local IOTF Criteria.

**Additional file 2: Supplementary Table 2.** Baseline characteristics’ difference between Study subjects (follow-up group) and lost to follow-up subjects.

## Data Availability

The datasets used and/or analyzed during the current study are available from the corresponding author on reasonable request.

## Abbreviations

CIMT: carotid intima media thickness
WHO: world health organization
CDC: center for disease control and prevention
IOTF: international obesity task force
NHANES: National Health and Nutrition Examination Survey
BMI: body mass index
LMS: Lambda-Mu-Sigma
TLGS: Tehran Lipid and Glucose Study
WC: waist circumference
FPG: fasting plasma glucose
TC: total cholesterol
HDL-C: high-density lipoprotein cholesterol
LDL-C: low density lipoprotein Cholesterol
TG: triglyceride
CV: coefficients of variation
LCCA: left common carotid artery
ICC: interclass correlation coefficient
AIC: Akaike’s information criterion
