# Supplementary material for: Comparison analysis of childhood body mass index cut-offs in predicting adulthood carotid intima media thickness: Tehran lipid and glucose study
Source: BMC Pediatr. 2021 Nov 6;21:494. doi: 10.1186/s12887-021-02963-y (PMC8571836; doi:10.1186/s12887-021-02963-y)
Supplement: Supplementary file 1 — Additional file 1: Supplementary Table 1. Cut-Off Values for BMI for Overweight and Obesity According to Local IOTF Criteria. [file 12887_2021_2963_MOESM1_ESM.docx]

| **Supplementary Table 1**. Cut-Off Values for BMI for Overweight and Obesity According to Local IOTF Criteria | | | | |
| --- | --- | --- | --- | --- |
| **Age** (years) | **Boy** | | **Girl** | |
|  | **Overweight** | **Obese** | **Overweight** | **Obese** |
| **2** | 15.96 | 17.35 | 15.71 | 17.65 |
| **3** | 15.96 | 17.35 | 15.89 | 17.65 |
| **4** | 16.20 | 17.68 | 16.07 | 18.14 |
| **5** | 16.46 | 18.03 | 16.27 | 18.63 |
| **6** | 16.73 | 18.41 | 16.53 | 19.13 |
| **7** | 17.03 | 18.83 | 16.92 | 19.69 |
| **8** | 17.37 | 19.31 | 17.47 | 20.36 |
| **9** | 17.78 | 19.87 | 18.17 | 21.17 |
| **10** | 18.24 | 20.51 | 19.00 | 22.10 |
| **11** | 18.78 | 21.25 | 19.97 | 23.15 |
| **12** | 19.37 | 22.07 | 21.00 | 24.32 |
| **13** | 20.03 | 22.99 | 22.01 | 25.52 |
| **14** | 20.73 | 23.99 | 22.91 | 26.65 |
| **15** | 21.48 | 25.07 | 23.69 | 27.65 |
| **16** | 22.25 | 26.23 | 24.29 | 28.50 |
| **17** | 23.04 | 27.46 | 24.76 | 29.17 |
| **18** | 25.00 | 30.00 | 25.00 | 30.00 |
