# Supplementary material for: Comparison analysis of childhood body mass index cut-offs in predicting adulthood carotid intima media thickness: Tehran lipid and glucose study
Source: BMC Pediatr. 2021 Nov 6;21:494. doi: 10.1186/s12887-021-02963-y (PMC8571836; doi:10.1186/s12887-021-02963-y)
Supplement: Supplementary file 2 — Additional file 2: Supplementary Table 2. Baseline characteristics’ difference between Study subjects (follow-up group) and lost to follow-up subjects. [file 12887_2021_2963_MOESM2_ESM.docx]

| **Supplementary Table 2.** Baseline characteristics’ difference between Study subjects (follow-up group) and lost to follow-up subjects | | | |
| --- | --- | --- | --- |
|  | **Follow-up Group** | **Lost to Follow-up Group** | ***P*-Value** |
| **Male** (%) | 665 (51.7) | 742 (48.7) | 0.116 |
| **Age** (years) | 10.9±4.1 | 11.4±4.4 | <0.001 |
| **Weight** (kg) | 40.4±17.6 | 42.3±19.3 | <0.001 |
| **BMI** (kg /m^2^) | 18.7±4.3 | 19.0±4.5 | 0.020 |
| **WC** (cm) | 64.5±11.2 | 65.6±12.5 | <0.001 |
| **Abdominal obesity** (%) | 154 (13.9) | 225 (16.5) | 0.082 |
| **SBP** (mmHg) | 104.0±11.7 | 104.8±12.2 | 0.213 |
| **DBP** (mmHg) | 70.3±9.6 | 71.2±9.5 | 0.789 |
| **Hypertension** (%) | 203 (16.2) | 243 (16.0) | 0.378 |
| **FPG** (mg⁄dl) | 87.4±8.2 | 87.3±12.2 | 0.179 |
| **TC** (mg/dl) | 169.9±31.6 | 168.3±32.6 | 0.980 |
| **TG** (mg⁄dl)**‡** | 93 (69-126.3) | 89 (67-124.8) | 0.182 |
| **HDL-C** (mg/dl) | 44.2±10.7 | 43.8±10.2 | 0.410 |
| **LDL-C** (mg/dl) | 104.4±28.0 | 103.6±28.2 | 0.477 |
| **Family history CVD** (%) | 47 (3.7) | 85 (5.3) | 0.031 |
| HDL-C, high-density lipoprotein cholesterol; LDL-C, low-density lipoprotein cholesterol; SBP, systolic blood pressure;  DBP, diastolic blood pressure; CIMT, Carotid Intima Media Thickness.  Data are given as the mean (SD) or median (IQ 25–75) unless otherwise indicated (‡). | | | |
